# Outcome Following the Treatment of Ventriculitis Caused by Multi/Extensive Drug Resistance Gram Negative Bacilli; *Acinetobacter baumannii* and *Klebsiella pneumonia*

**DOI:** 10.3389/fneur.2018.01174

**Published:** 2019-01-23

**Authors:** Sajan Pandey, Lei Li, Xian Yu Deng, Da Ming Cui, Liang Gao

**Affiliations:** ^1^Neurosurgery Department, Shanghai Tenth People's Hospital, Tongji University, Shanghai, China; ^2^Shanghai Tenth People's Hospital, Tongji University, Shanghai, China

**Keywords:** multi drug resistance ventriculitis, extensive drug resistance ventriculitis, ventricular lavage, intraventricular colistin therapy, external ventricular drain

## Abstract

**Introduction:** CNS ventriculitis is a serious complication following an intracranial insult that demands immediate treatment with broad-spectrum antibiotics in a critical care setting. Infections due to multi/extensive drug resistance (MDR/XDR) microorganisms are very challenging, which may demand an additional approach to the ongoing practice; intravenous and intraventricular administration of antibiotics.

**Aim:** To study the efficacy and safety of thorough ventricular irrigation followed by daily intraventricular antibiotic administration in patients with MDR/XDR ventriculitis.

**Materials and Methods:** A retrospective analysis was done on 19 inpatients with ventriculitis caused by *Acinetobacter baumannii* (AB) or *Klebsiella pneumonia* (KP), at Shanghai Tenth People's Hospital from January 2016 to October 2017. We reviewed our experience; the role of thorough ventricular irrigation with Colistin mixed normal saline, followed by intraventricular Colistin therapy. Treatment outcomes were evaluated based on the clinical symptoms, Cerebro-Spinal Fluid (CSF) culture, laboratory findings and complications.

**Results:** A total of 19 patients were included (15 males and 4 females), with a mean age in years of 51, which ranged from 18–67. Fourteen patients had *Acinetobacter baumannii* (AB) and 5 had *Klebsiella pneumoniae* (KP). The average CSF sterilization period following ventricular irrigation and intraventricular Colistin was 6 days. Sixteen patients (84%) were cured, and 3 patients (15%) died during the course of the treatment.

**Conclusion:** In addition to Intraventricular Colistin, thorough ventricular irrigation could increase the cure rate up to 84% in patients suffering from MDR/XDR CNS ventriculitis.

## Introduction

Central nervous system infections are caused by wide range of microorganisms. It presents with different clinical syndromes such as meningitis, encephalitis, brain abscess, and ventriculitis, etc. Morbidity and mortality of these patients depends on several factors: type of an organism involved and its sensitivity to the available antibiotics, location as-well as severity of infections such as localized brain abscess or ventriculitis. Prompt recognition and management of these patients is absolutely crucial for significant reduction on morbidity and mortality rates.

Health care associated intracranial infections are often encountered in neurosurgical patients (1). According to the CDC, it is defined as a localized or systemic condition resulting from an adverse reaction to the presence of an infectious agent(s) or its toxin(s), without any evidence that the infection was present or incubating at the time of admission (2), and can be seen within 48 h to a week following a hospital discharge (3).

The incidence of health care associated intracranial infection varies according to the type of insults. Two large series studies reported its incidence ranging from 0.3 to 1.5% in patients who had a neurosurgical procedure (4, 5). Another study indicated that 3 to 29% of sub-arachnoid hemorrhage (SAH) patients suffered from bacterial ventriculitis, mainly those who received cerebrospinal fluid (CSF) diversion via a catheter (6–9). Similarly, a study done on 520 patients, admitted following a traumatic brain injury (TBI), showed that 6.54% suffered from an intracranial infection (10). The study also revealed a significant correlation between intracranial infection and a CSF leak or a drainage (either ventricular or lumbar) and number of craniotomies.

As compared to community acquired intracranial infections, the distribution of causative organisms are different in health care associated infections, which are associated with normal flora of skin and nasopharynx such as *Streptococcus pneumonia, staphylococci*, and gram-negative organism (11, 12). Treatment targeted to these organisms has been complicated by the appearance of resistant gram negative bacteria like *Acinetobacter baumannii* and *Klebsiella pneumonia*, which are resistant to many extended spectrum and fourth generation cephalosporin, carbapenems and aminoglycosides antibiotics (13). Studies done in Ventriculitis caused by multiple drug resistant bacteria, following a neurosurgical procedures, shows higher morbidity and mortality from 71.4 to 72.7%, mainly due to the drug resistance and inadequate penetration of antibiotics from the blood brain barrier (BBB) (14, 15). Another study by Chen et al. done on 13 AB infected patients with carbapenem resistant organism, demonstrated 30% mortality rates (16).

Fortunately, AB is sensitive to polymyxins, but the challenge of polymyxin remains to be its penetration through the BBB. Which is very low requiring higher dose of intravenous polymyxin. Therefore, the intraventricular/intrathecal route has been chosen in several published papers in-order to meet the concentration of polymyxin required to combat the bacteria (17–21).

Until today there is not enough evidence to develop an exact guideline describing polymyxins dose/duration for patients with intracranial infection by AB. We encountered 19 patients with *A. baumannii* and *K. pneumoniae* and treated them by ventricular irrigation during the EVD placement, followed by intraventricular polymyxin and shared our experience in this paper.

## Materials and Methods

Data was collected from the patients admitted from the Neurological Intensive Care Unit (NICU) at the Department of Neurosurgery, Shanghai Tenth People's Hospital (Shanghai, China), between 1st January 2016 and 1st October 2017.

### Inclusion Criteria

Patients were selected with (i) clinical and imaging evidence suggestive of ventriculitis (ii) microbiological evidence of CNS infection (iii) isolation of MDR/XDR gram-negative bacilli from the CSF (iv) thorough ventricular irrigation with Colistin mixed normal saline in the operating room and administration of intraventricular Colistin via the IVT/IHT route in the ICU.

#### Imaging Criteria

Findings from Computer tomography (CT) and Magnetic resonance (MR) Imaging that showed irregular ventricular debris, Hyperintense to CSF on T1 weighted images and hypointense to CSF on T2 weighted images, or periventricular hyperintense signal, were considered suggestive of ventriculitis (22).

### Patients Information

The following data were recorded retrospectively: age, sex, underlying medical conditions, previous infections before the symptoms of CNS infection onset, current diagnosis, presence of foreign bodies, prior antibiotic use, days from an admission to the diagnosis of the CNS infection, culture results, susceptibility to antibiotics, concomitant infections except for CNS infection, time from CNS infection onset to ventricular irrigation, Colistin doses, and duration of intravenous (IVT)/intrathecal (ITH) Colistin treatment, concurrent antimicrobial regimens, days for sterilization of CSF, toxicity to Colistin, and outcomes (Table 1).

**Table 1:** Patient distribution in terms of age, sex, prior hospitalization before the ventriculitis onset in days, isolated microorganism and resistance in term of Multidrug resistance (MDR) and extensive drug resistance (XDR), concomitant infection, prior antibiotics use and foreign body.

**Patient characteristics**	**Mean ±*SD* (range) or *n* (%)**
Age	51.0 ± 13.8 (18–67)
Male sex	15 (78.9)
Prior hospitalization before ventriculitis onset(days)	20.0 ± 10.6 (7–49)
**ISOLATED ORGANISM**	
*A. baumannii*	14 (MDR 12, XDR 2)
*K. pneumoniae*	5 (MDR 4, XDR 1)
**CONCOMITANT INFECTION**	
Pneumoniae	17
Prior antibiotic use	19 (100)
**FOREIGN BODY**	
EVD	12
VP-Shunt	3
ICP monitor	1
Lumbar drainage	1

### External Ventricular Drainage Description

Medtronic™ I external ventricular system was used for the drainage. Tube was inserted 5 cm from the skin to bone and 5.5 cm from the bone to the ventricle.

### Specimen Collection and Lavage

About 30 ml of purulent cerebrospinal fluid was aspirated and sent for a microbiological culture prior to the ventricular lavage. Intraventricular lavage was done by adding 10 mg Colistin per 500 ml normal saline. The intraventricular lavage was administered via an external ventricular drainage tube and continued until the lavage fluid became transparent (or clear).

### Microbiological Assays

Clinical isolation of bacteria and antimicrobial susceptibility testing were performed according to methods defined by the Clinical and Laboratory Standards Institute (CLSI) (23). An organism was classified as MDR or XDR according to the regime of an international expert proposal for interim standard definitions for acquired resistance (24).

### Intraventricular Colistin Therapy

The IVT/ITH Colistimethate sodium (Colistin; Hong Kong, China) was administered at a dose of 100,000 IU (International Unit) every 12–24 h. Doses were administered according to the initial CSF white blood cell (WBC) count. If the WBC counts were higher than 5,000 per micro liter, 100,000 IU Colistin was given q12 hourly, but if the count was < 5000 per micro liter, they received 100,000 IU Colistin q24 hourly. After the intraventricular Colistin administration, CSF drainage was stopped for 2 h.

### Outcome

The outcome was defined as a cure if a patient fulfilled the following criteria: (i) resolution of symptoms and signs of CNS infection (ii) no subsequent need for additional antimicrobial therapy; (iii) eradication of MDR/XDR Gram-negative bacilli in subsequent cultures of CSF.

### Data Analysis

Data was recorded and analyzed using Microsoft office Excel 2016. The study was approved by the Ethics Committee of Shanghai Tenth People's Hospital.

## Results

According to our data, 19 patients were identified infected, 5 of which were due to *K. pneumonia*, and 14 of which were due to *A. baumannii*. Among 19 patients, 3 were XDR (1 *K. pneumoniae* and 2 *A. baumannii*) and 16 were MDR (4 *K. pneumoniae* and 12 *A. baumannii*). All critically ill patients with MDR/XDR gram-negative bacilli infections had received empirical antimicrobial therapy from their respective referral health care centers.

All 19 patients were treated with intraventricular lavage and IVT Colistin based on the available culture results. On average, around 1,000 ml normal saline mixed Colistin (10 mg/500 ml normal saline) was required to irrigate ventricles to acquire colorless CSF. Upon CSF analysis, 3 *(A. baumannii)* out of 19 patients had a WBC count of < 5,000 cells per microliter, and these three patients were given q24 hourly Colistin whereas rest of the 16 received q12 hourly. A polymicrobial infection due to *A. baumannii* and *S. epidermidis* was identified in one of these patients. Patient characteristics and bacterial species of MDR/XDR are presented in Table 1 and their clinical features are presented in Table 2.

**Table 2:** Ventriculitis case characteristics.

**Case**	**Age/sex**	**GCS prior to ventricular lavage**	**Underlying conditions**	**CSF isolates**	**Foreign body**	**Susceptibility/sensitivity of organism from CSF**	**Concomitant infection**	**Time from symptoms onset to ventricular irrigation**	**Intraventricular Colistin duration**	**Time to CSF sterilization**	**Outcome/GCS**
1	51, F	8	Cerebral hemorrhage	*Acinetobacter baumannii*	EVD	Susceptible to colistin; Intermediate to Cefoperazone sulbactam sodium + Minocycline	Pneumoniae (*Acinetobacter baumannii*)	11	36	4	Cure/15
2	61, M	7	Posterior communicating aneurysm rupture with SAH	*Klebsiella pneumoniae*	EVD	Susceptible to colistin	Pneumoniae (*Klebsiella pneumoniae* + *Pseudomonas aeruginosa*)	2	3	6	Cure/15
3	65, M	5	Brain stem bleeding/Hydrocephalus	*Klebsiella pneumonia*	VP-Shunt	Susceptible to colistin; Intermediate to Fosfomycin + tigercycline	Pneumoniae + Bacteremia(*Klebsiella pneumoniae*)	4	6	/	Dead/3
4	46, M	5	Traumatic brain injury	*Acinetobacter baumannii*	EVD	Susceptible to colistin + Minocycline	Pneumoniae (*Klebsiella pneumoniae + Acinetobacter baumannii*)	3	32	29	Cure/13
5	53, M	6	Traumatic brain injury	*Acinetobacter baumannii*	EVD	Susceptible to colistin; Intermediate to tigercycline	/	13	31	5	Cure/15
6	47, M	6	Traumatic brain injury	*Acinetobacter baumannii*	EVD	Susceptible to colistin; Intermediate to Levofloxacin + Minocycline	Pneumoniae (*Acinetobacter baumannii*)	6	18	4	Cure/13
7	39, M	7	Traumatic brain injury	*Acinetobacter baumannii*	None	Colistin; Intermediate to Levofloxacin	Pneumoniae (*Acinetobacter baumannii*)	3	30	7	Cure/14
8	49, M	8	Traumatic brain injury	*Staphylococcus epidermidis + Acinetobacter baumannii*	VP-Shunt	Susceptible to colistin; Intermediate to tigercycline	Pneumoniae (*Pseudomonas aeruginosa + Neisseria meningitidis*)	9	23	6	Cure/15
9	62, M	5	Cerebral hemorrhage	*Klebsiella pneumoniae*	ICP monitor	Susceptible to colistin+Fosfomycin; Intermediate to Minocycline	Pneumoniae (*Klebsiella pneumoniae*)	/	3	/	Dead/3
10	60, M	7	Cerebral hemorrhage	*Acinetobacter baumannii*	None	Susceptible to colistin; Intermediate to Levofloxacin	Pneumoniae (*Acinetobacter baumannii + Enterobacter cloacae*)	10	21	4	Cure/15
11	65, F	6	Aneurysmal	*Acinetobacter baumannii*	EVD	Susceptible to colistin; Intermediate to tigercycline	Pneumoniae (*Acinetobacter baumannii+Stenotrophomonas maltophilia + Staphylococcus aureus*)	7	36	3	Cure/10
12	63, M	9	Cerebellar hemorrhage, hydrocephalus, intestinal obstruction	*Klebsiella pneumoniae*	VP-Shunt	Susceptible to colistin+Amikacin	Pneumoniae (*Klebsiella pneumoniae+Pseudomonas aeruginosa*)	11	13	2	Cure/14
13	23, F	7	Cerebral hemorrhage	*Klebsiella pneumoniae*	EVD	Susceptible to colistin+ Sulfamethoxazole+Minocycline	Pneumoniae (*Klebsiella pneumoniae*)	7	14	6	Cure/15
14	51, M	9	Cerebral hemorrhage	*Acinetobacter baumannii*	EVD	Susceptible to colistin; Intermediate to Levofloxacin+Cefoperazone sulbactam sodium	Pneumoniae (*Klebsiella pneumoniae + Acinetobacter baumanni*i)	14	11	3	Cure/15
15	37, F	9	Cerebral hemorrhage	*Acinetobacter baumannii*	EVD	Susceptible to colistin	/	5	27	4	Cure/14
16	54, M	8	Cerebral hemorrhage	*Acinetobacter baumannii*	Lumbar drainage	Susceptible to colistin; Intermediate to Cefoperazone sulbactam sodium	Pneumoniae (*Klebsiella pneumoniae + Acinetobacter baumannii*)	14	31	5	Cure/15
17	58, M	5	Cerebral hemorrhage	*Acinetobacter baumannii*	None	Susceptible to colistin+SMZCO	Pneumoniae (*Acinetobacter baumannii + viridans streptococci*)	28	6	/	Dead/3
18	67, M	6	Cerebral hemorrhage	*Acinetobacter baumannii*	EVD	Susceptible to colistin;Intermediate to Levofloxacin+Cefoperazone sulbactam sodium	Pneumoniae (*Klebsiella pneumoniae + Pseudomonas aeruginosa*)	7	21	9	Cure/10
19	18, M	7	Traumatic brain injury	*Acinetobacter baumannii*	EVD	Susceptible to colistin	Pneumoniae (*Klebsiella pneumoniae + Pseudomonas aeruginosa*)	21	39	8	Cure/12

*Number of cases with underlying conditions before suffering from ventriculitis, GCS prior and after the colistin thereapy, type of foreign body involved, antibacterial susceptibility to organisms, presence of concomitant infection, time from symptoms onset to ventricular irrigation, intraventricular colistin therapy in days, time to sterilize CSF following ventricular irrigation and therapy and the final outcome after the treatment. Isolated organisms in all cases were resistant to carbapenem*.

Of the 19 patients with an MDR/XDR infection (15 males and 4 females), patient age ranged from 18 to 67 years old with an average of 51.0 years. The underlying condition for the ventriculitis was hemorrhage in 13 patients (68.4%) and traumatic brain injury in 6 patients (31.6%). Out of 19, one patient had a previous pulmonary infection before suffering from the ventriculitis. Twelve patients (63.2%) had EVD, 3 patients (15.8%) had VP shunt, 1 patient (5.3%) had lumbar drain, and 3 patients (15.8%) had no drainage system. Twelve patients (63.2%) were treated with Vancomycin plus Meropenem whereas 7 patients (36.8%) were treated with several antibiotics: Meropenem, Tigecycline, Daptomycin, Linezolid, Sulfamethoxazole, Cefaperazone, Sulbactam Sodium, in combination as indicated by previous culture and sensitivity reports. The average number of days of hospitalization before the development of ventriculitis was 19 days, which ranged from 7 to 49 days. *AB* alone was isolated in 13 patients (68.4%), *KP* alone was isolated in 5 patients (26.3%) and combined *AB plus Staphylococcus epidermis* were isolated in 1 patient (5.3%). Sensitivity tests for these organisms showed 100% sensitivity to Colistin. Seventeen patients (89.5%) showed concomitant pulmonary infection (10 with *K. pneumoniae* and 7 with *A. baumannii*), and 2 patients (10.5%) didn't have a concomitant infection. The average time of ventricular lavage with Colistin mixed normal saline (10 mg CMS in 500 ml NS) following the onset of ventriculitis symptoms was 7 days, which ranged from 2 to 28 days. Other intravenous antibiotics received for various ongoing infections during the course were; Tigecycline, Sulbactam sodium, Rifampicin, Fosfomycin, Cefaperazone, Levofloxacin, Meropenem, Linezolid, Vancomycin, Amikacin, Cefepime, Cotrimoxazole.

Among 19 patients, 3 patients (15.8%) died during the course of the treatment, 2 patients due to *K. pneumonia* and 1 patient due to *A. baumannii*. CSF analysis until the end was unsterile for all 3 patients. But after CSF analysis following the irrigation and intraventricular course, 16 out of 19 patients (84.2%) showed sterile CSF. The average duration to achieve sterile CSF among 16 patients was 6.56 days which ranged from 2 to 29 days. Out of 19, 16 patients who made it to sterile CSF stage were cured and didn't relapse following the treatment course. None of the patients suffered from side effects related to Colistin. On average all patients received intraventricular Colistin for 21 days, which ranged from 3 to 39 days.

All patients received IVT CMS therapy as part of the treatment for the MDR infection.

## Discussion

Multiple drug resistance intracranial infection due to organisms *A. baumannii* and *K. pneumonia* brings significant morbidity and mortality along with it (14–16) demanding extra precautions and additional therapeutic approaches to minimize the consequences following these infections. Factors such as increasing resistance to most of the antibiotics and low penetration through the BBB to counter these organisms, are thought to be responsible for higher morbidity/mortality. As a result, many studies have chosen the intraventricular or intrathecal route to administer the drug directly, in order to achieve the necessary steady state to combat these organisms (17, 18, 20, 21). Although Meropenem is the recommended empirical therapy for intracranial infection caused by *Acinetobacter* (25, 26), Carbapenem is becoming increasingly ineffective due to the development of rapidly increasing resistance (40% increase) (27).

Fortunately, Polymyxin, an old antibiotic from 1950s, seems to be effective against these multi drug resistance strains (28). Its intravenous use poses some limitations which are primarily due to its poor ability to penetrate through the BBB and are often assumed due to the higher molecular weight and polycationic structure of polymyxins (29). As a result, intravenous Colistin did not achieve adequate CSF drug levels to counter MDR pathogens (30). Previous studies and the studies done in 2004 by the infectious disease society of America suggested that the intraventricular use of Colistin 0.125 MIUs (million international unit) is needed to achieve a sufficient steady state, specifically for groups where eradication is otherwise difficult (17–21).

Administration of intrathecal or intraventricular antibiotics requires special tools such as EVD or Ommaya attached to it, but we should not overlook the fact EVD itself is a foreign body which could serve as a potential source of an infection.

External ventricular drains are routinely used in the neurocritical care for both therapeutic and diagnostic purposes. Despite having multiple benefits and the use of prophylactic antibiotics (Intravenous Cefuroxime 1,500 mg half hour before the insertion of EVD and Piperacillin-tazobactam 2,500 mg q12 hourly for 3 days), EVD poses an increased risk of intracranial infection ranging from 0 to 22% (31). The appearance of gram negative bacteria like *A. baumannii* and *K. pneumonia* can be very challenging as they are increasingly resistant to many extended spectrum and fourth generation cephalosporin, carbapenems, and aminoglycosides antibiotics (13). Taking this into consideration, some authors suggest whenever possible, especially in a situation like failed primary therapy, intrathecal administration should be done by repeat lumbar puncture on an everyday basis, thereby removing EVD and lumbar drain to avoid a foreign body being attached to the system (17).

The IDSA guidelines recommend strong, or moderate for the use of meropenem for strains that demonstrate carbapenem resistance, and recommends Colistimethate sodium or polymyxin B agents by intravenous and intraventricular route but it doesn't show the benefit of thorough ventricular lavage with the colistin before intraventricular and intravenous colistin administration (31). We approached our patients via removing the shunts (if present previously) and installed a new drainage system, as the old catheters were already infected and could act as a bacterial reservoir. Furthermore, we could not deny the possibility of the catheter clogging as they have already been in contract with thick viscous fluid. Catheter clogging in the middle of the treatment would certainly hamper intraventricular administration of Colistin and the CSF drainage. In addition to intraventricular or intrathecal Colistin administration, we did a thorough ventricular irrigation with Colistin (10 mg CMS per 500 ml normal saline) in an operating room immediately after the placement of an EVD. The notion behind the thorough lavage until the lavage fluid appears clear was to;

(i) Reduce inflammation
(ii) Enhance CSF flow
(iii) Enhance adequate antibiotics perfusions.

We assumed that reducing the amount of pus in the CSF would also reduce the number of organisms and inflammatory cytokines. Pus mixed CSF is more viscous which might cause impaired CSF circulation simply due to high viscosity or blockage of the interventricular foramens, bringing viscosity close to the viscosity of normal CSF which would help to bring CSF circulation close to its normal level. This would not only help to clear the CSF but also help to circulate antibiotics throughout the entire CNS efficiently. Several studies done in intracranial infection have shown the benefit of ventricular lavage in terms of morbidity and mortality (31–34). During the intraventricular drug administration, the shunts were closed for 2 h following the administration of Colistin to prevent immediate drainage of the administered antibiotics, so that the amount required to counter the organism would remain within the CSF circulation.

Our study suggests combined ventricular irrigation with intraventricular/intrathecal Colistin administration brings a superior outcome with respect to duration of early sterilization of CSF, morbidity, and mortality, as compared to intrathecal/intraventricular administration with or without IV Colistin administration (21, 35). In one of the largest reviews of the use of IVT Colistin in neurosurgical patients, Karaiskos et al. reviewed 81 cases of multidrug-resistant and extremely drug-resistant *A. baumannii* ventriculitis/meningitis, in which IVT Colistin was administered, and these investigators reported successful clinical and bacteriologic outcome in 89% of patients (72/81) (18). Our result demonstrated an 84% success rate in terms of cure, which is higher than previous published cases and it exclusively contains patients suffering from XDR/MDR Ventriculitis, whereas previous studies contained other forms of CNS infection such as meningitis. Our study suggests not only a higher cure rate in ventriculitis patients, but it also showed early CSF sterilization(6 days on average, ranging from 2 to 29 days) as compared to an average of 21 days shown by a previous study on ventriculitis by De Bonis et al. (21). On average, all patients received intraventricular Colistin for 21 days, which ranged from 3 to 39 days. A lower value of 3 days was observed as one of the patient died on the 3rd day, but the other two patients died on the 6th day, following the ventricular irrigation and daily intraventricular Colistin therapy. Patients with a higher WBC count (more than 5000 per micro liter) received q12 hourly 100,000 IU intraventricular Colistin, assuming higher antibiotic concentration would be required to counter the organisms. Whereas, the other group with a WBC count < 5,000 per micro liter were given q24 hourly 100,000 IU intraventricular Colistin. The outcome for both groups was promising. Despite having a higher cure rate and shorter mean duration to CSF steralization we should not forget the existing variation between these subjects which is high, specifically in terms of the CSF steralization period ranging from 2 to 29 days. The observed variation could be due to several factors such as the patient's immune function, GCS, concomitant infection, other comorbid conditions, nutritional status, and perhaps many other factors. So, despite all the efforts to overcome these possible associating factors in the critical care setting, we should not undermine the fact that every patient shows different response to a similar theraputic approach.

## Conclusion

According to our experience in a small group of patients suffering from MDR/XDR related ventriculitis, the outcome following a thorough ventricular lavage followed by the intraventricular/intrathecal Colistin administration could be considered superior to the intraventricular Colistin administration. Though the results are promising, due to the small sample size and lack of a control group it would be too early and unreliable to draw a conclusion. Therefore, there is a demand for further studies using a larger sample size and a control group.

## Author Contributions

SP wrote the manuscript, contributed to data collection and analysis. LL contributed to manuscript preparation and data collection. XD contributed to data collection. DC and LG reviewed the manuscript.

### Conflict of Interest Statement

The authors declare that the research was conducted in the absence of any commercial or financial relationships that could be construed as a potential conflict of interest.

## Abbreviations

CSF: Cerebrospinal fluid
WBC: White blood cells
IVT/ITH, intravenous: intrathecal
SAH: Sub arachnoid hemorrhage
TBI: Traumatic Brian Injury
EVD: External ventricular drainage
LD: Lumbar drainage
VP shunt: Ventriculo-peritoneal shunt
*AB*: *Acinetobacter baumannii*
*KP*: *Klebsiella pneumonia*
MDR: Multiple drug resistance
XDR: Extensive Drug Resistance
CT: Computed Tomography
MRI: Magnetic Resonance Imaging
IU: International Unit
CLSI: Clinical and Laboratory Standards Institute.
